# Treatment Patterns and Prognostic Nomograms for Overall Survival and Hepatic Progression-Free Survival in Unresectable Colorectal Liver Metastases Treated with Drug-Eluting Bead Chemoembolization: A Single-Center Study

**DOI:** 10.3390/curroncol33090497

**Published:** 2026-08-22

**Authors:** Ketong Wu, Haiyang Chen, Dan Li, Yuan Wan, Weiyao Li, Bo Zhang

**Affiliations:** 1 Department of Interventional Radiology, The Sixth Affiliated Hospital, Sun Yat-sen University, Guangzhou 510655, China; wuketong3@mail.sysu.edu.cn (K.W.); lidan259@mail.sysu.edu.cn (D.L.); wany26@mail2.sysu.edu.cn (Y.W.); 2 Biomedical Innovation Center, The Sixth Affiliated Hospital, Sun Yat-sen University, Guangzhou 510655, China; chenhy76@mail.sysu.edu.cn; 3 Department of Radiotherapy, The Sixth Affiliated Hospital, Sun Yat-sen University, Guangzhou 510655, China

**Keywords:** colorectal liver metastases, chemoembolization, therapeutic, nomograms, survival analysis, carcinoembryonic antigen, prognosis

## Abstract

**(1) Background:** Drug-eluting bead transarterial chemoembolization (DEB-TACE) is increasingly used for unresectable colorectal liver metastases (CRLM), yet individualized prognostic tools are lacking. We developed and internally validated nomograms predicting overall survival (OS) and hepatic progression-free survival (hPFS). **(2) Methods:** In this single-center retrospective cohort, reported per the TRIPOD guideline, OS and hPFS were estimated by Kaplan–Meier methods, and independent predictors from multivariable Cox regression were assembled into nomograms. Internal validation combined 1000-sample bootstrap optimism-corrected concordance indices (C-index), a uniform shrinkage factor, a bootstrap calibration slope, and a LASSO–Cox sensitivity analysis. **(3) Results:** Among 63 patients (44 deaths; 42 intrahepatic-progression events), median OS was 10.9 months and median hPFS was 5.8 months. Independent OS predictors were baseline CEA, high liver tumor burden (≥10 lesions), CEA decline (protective), and second-line-or-beyond interventional therapy (corrected C-index: 0.796). Independent hPFS predictors were high liver tumor burden, CEA decline, and age (corrected C-index: 0.718). Nomogram-defined high-risk groups had markedly shorter OS (5.3 vs. 22.8 months) and hPFS (3.3 vs. 8.6 months; both *p* < 0.001). Grade ≥3 toxicity occurred in 6%. **(4) Conclusions:** In real-world DEB-TACE-treated CRLM, liver tumor burden and CEA dynamics dominated prognosis; the internally validated nomograms provide individualized estimates and risk stratification, pending external validation.

## Simple Summary

About half of people with colorectal cancer develop liver metastases, and for many the disease cannot be removed surgically. One option delivers chemotherapy directly into the liver tumors using drug-eluting beads (DEB-TACE), but how long individual patients benefit varies widely, and clinicians have lacked simple tools to estimate prognosis. In this single-center study of 63 such patients, two routinely available measures—namely, the number of liver tumors and the blood level of carcinoembryonic antigen (CEA) together with its early change after treatment—captured much of the variation in outcome. We combined these factors into easy-to-use charts (nomograms) that translate them into individualized estimates of overall survival and of the time before the liver disease progresses, which sorted patients into risk groups with markedly different outcomes. These tools could help clinicians discuss prognosis, identify higher-risk patients who might benefit from more intensive systemic therapy or closer monitoring, and support shared decision-making. Because the study was small and single-center, the tools require validation in larger, independent cohorts before routine use.

## 1. Introduction

Colorectal cancer is among the most common malignancies worldwide, and the liver is the dominant site of metastatic spread [1]. Approximately half of people with colorectal cancer develop liver metastases during the course of the disease, and hepatic involvement is the principal determinant of survival [2]. Although surgical resection offers the only realistic prospect of cure, most present with unresectable disease owing to multifocal hepatic involvement, an inadequate future liver remnant, extrahepatic spread, or comorbidity [3]. For these patients, locoregional therapies that concentrate cytotoxic exposure within the liver have become an important component of multidisciplinary management [4,5].

Drug-eluting bead transarterial chemoembolization (DEB-TACE) delivers cytotoxic drug-loaded microspheres into the tumor-feeding arteries, combining sustained intratumoral drug release with an embolic, ischemia-inducing effect [6,7,8,9,10]. Hepatic arterial infusion chemotherapy (HAIC) instead delivers high-dose chemotherapy by arterial perfusion [11,12,13]. In real-world practice, these modalities are not mutually exclusive: DEB-TACE is frequently combined with HAIC or with continued systemic therapy, producing a spectrum of treatment patterns that does not map neatly onto the controlled comparisons reported in clinical trials.

Existing prognostic frameworks for colorectal liver metastases are dominated by surgical and population-registry models. Widely used tools such as the Fong clinical risk score and subsequent nomograms were developed in patients undergoing hepatic resection, and large nomograms derived from the Surveillance, Epidemiology, and End Results database describe heterogeneous, mostly resected or systemically treated populations [14]. These instruments do not capture the distinct biology and treatment exposures of patients managed with arterially directed therapy, in whom intrahepatic tumor burden, vascular delivery, and early biochemical response may carry different prognostic weight [15,16]. As a result, a clinician counseling a patient before or during DEB-TACE has no validated, population-appropriate tool to estimate that individual’s likely overall or hepatic-specific outcome.

Two practical problems follow. First, the relative contribution of each treatment combination is difficult to isolate outside randomized settings, and small or imbalanced subgroups preclude reliable head-to-head survival comparisons. Second, and more importantly for the individual, clinicians lack validated tools to estimate prognosis within the DEB-TACE-treated population, where outcomes vary widely. Prognostic models that integrate readily available baselines and early-response variables could help to stratify risk, inform expectations, and guide the intensity of subsequent therapy [17]. We therefore described treatment patterns in a real-world cohort, and developed and internally validated nomograms predicting OS and hPFS. To our knowledge, this is the first study to derive and internally validate nomograms for both overall survival and hepatic progression-free survival specifically in DEB-TACE-treated CRLM. By explicitly modeling intrahepatic progression—a directly actionable endpoint for locoregional therapy that has rarely been addressed—and by relying only on variables available at the point of care, the resulting tools are positioned to support prognostic assessment in the real-world combination setting that randomized trials do not capture, pending external validation.

## 2. Materials and Methods

### 2.1. Study Design and Population

**Figure 1:**
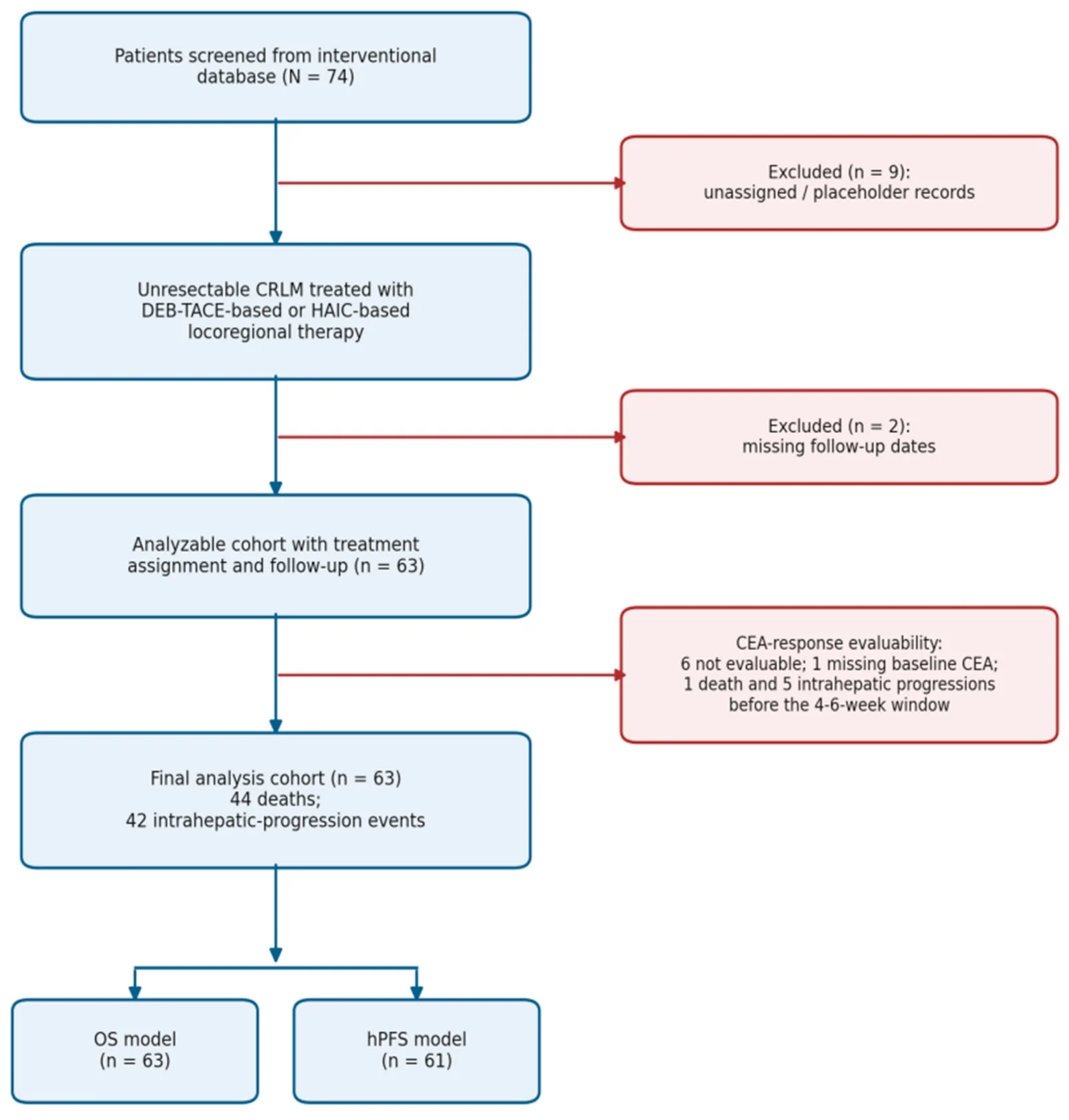
Flow diagram of patient selection from screening to the final analysis cohort.

This retrospective cohort study used a prospectively maintained single-center interventional radiology database. Eligible patients had histologically confirmed colorectal adenocarcinoma with radiologically documented unresectable liver metastases and had received DEB-TACE-based or HAIC-based locoregional therapy. Patients with incomplete treatment assignment or without evaluable follow-up were excluded. After exclusions, 63 patients with complete survival follow-up constituted the analyzable cohort (Figure 1). The study was approved by the institutional review board and conducted in accordance with the Declaration of Helsinki.

### 2.2. Treatment Protocols and Subgroups

DEB-TACE was performed under fluoroscopic guidance via superselective catheterization of the tumor-feeding hepatic arteries using irinotecan-loaded CalliSpheres or DC Bead microspheres (100–300 μm in diameter, loaded with 100 mg of irinotecan per vial), which were slowly delivered via a microcatheter under fluoroscopic guidance. The injection was continued until the predefined embolic endpoint of near-stasis or complete stasis of the tumor-feeding arterial branches was achieved. HAIC employed an oxaliplatin- or raltitrexed-based regimen infused through an arterial microcatheter. Treatment subgroups reflected real-world practice and comprised DEB-TACE plus HAIC, DEB-TACE monotherapy, DEB-TACE plus systemic chemotherapy, and HAIC monotherapy. Because the HAIC-monotherapy subgroup was small, between-subgroup survival differences were treated as descriptive and hypothesis-generating rather than as formal efficacy comparisons.

### 2.3. Endpoints and Variable Definitions

The co-primary endpoints were OS, defined from the first interventional procedure to death from any cause, and hPFS, defined from the first procedure to radiologically confirmed intrahepatic progression; patients without an event were censored at last follow-up. Tumor response was assessed by RECIST v1.1 [18]. CEA decline was defined as a reduction in serum carcinoembryonic antigen, measured at the first post-treatment evaluation (4–6 weeks after the index DEB-TACE procedure, concurrent with the first RECIST v1.1 imaging assessment), relative to the pre-treatment baseline value [19]. Patients without a paired baseline and follow-up CEA within this window were classified as not evaluable for CEA response. High liver tumor burden was defined as ten or more hepatic lesions. Adverse events were graded according to CTCAE v5.0 [20].

The ten-lesion threshold was pre-specified from the prior literature on hepatic tumor burden in colorectal liver metastases and was not optimized on the present dataset; an ordinal three-level burden analysis (low: 4 or fewer; intermediate: 5–9; high: 10 or more) was performed as a dose–response sensitivity check, which showed a graded association with both endpoints (OS hazard ratio per level: 1.66, 95% CI: 1.11–2.47, *p* = 0.013, C-index: 0.637; hPFS: 1.49, 95% CI: 0.98–2.28, *p* = 0.061, C-index: 0.581; Table S12), supporting the pre-specified ten-lesion dichotomy. Lesion counts were frequently recorded as ranges (e.g., “10+”), precluding exact continuous modeling.

***Intended Clinical Use:*** The nomograms are intended as a two-step tool. Before treatment, the baseline variables (baseline CEA, high liver tumor burden, and interventional treatment line for OS; high liver tumor burden and age for hPFS) provide a provisional estimate for counseling; because post-treatment CEA decline is not yet observable, it is genuinely unknown at baseline. We therefore recommend either using the baseline variables alone for pre-treatment risk estimation or presenting the full-model output explicitly as two conditional scenarios (“if CEA declines” versus “if CEA does not decline”), rather than defaulting to the non-declining state, which would systematically overestimate risk in patients who subsequently show a CEA response. At the first post-treatment assessment (4–6 weeks), the observed CEA response is entered to refine the estimate. The nomograms are, therefore, combined baseline and early-response tools rather than purely pre-treatment predictors.

***Handling of Early Events and Censoring:*** Patients who died or developed intrahepatic progression before the 4–6-week CEA assessment, and those without a paired baseline and follow-up CEA, were classified as not evaluable for CEA response and coded as non-declining (a conservative assignment); because classification as declining requires a documented paired measurement, no patient who was declining could be classified before surviving to the assessment. In the hPFS analysis, deaths without prior documented intrahepatic progression were censored at the date of death, with a competing-risks sensitivity analysis treating such deaths as a competing event. Two patients without any evaluable post-treatment hepatic imaging could not be assessed for intrahepatic progression and were therefore excluded from the hPFS analysis (hPFS analytic cohort, *n* = 61), while both were retained in the OS analysis (*n* = 63).

### 2.4. Statistical Analysis

The study was reported in accordance with the TRIPOD statement. Continuous variables are summarized as medians with ranges and categorical variables as counts and percentages. Survival was estimated by the Kaplan–Meier method. Candidate prognostic variables were screened by univariable Cox proportional-hazards regression; variables associated with the endpoint at *p* < 0.10, after consideration of collinearity and the available number of events, were entered into multivariable Cox models. To respect the events-per-variable constraint, final models were restricted to a parsimonious set of independent predictors (four for OS, three for hPFS; approximately 11 and 14 events per variable) [21]. Nomograms were constructed from the multivariable coefficients.

Because the sample was modest, internal validation was deliberately comprehensive and designed to detect and quantify overfitting. Discrimination was quantified by Harrell’s C-index, corrected for optimism using 1000 bootstrap resamples, and was additionally assessed by repeated 5-fold cross-validation (20 repetitions) reporting the mean C-index with standard deviations and 95% confidence intervals [22]. Overfitting was assessed by (i) a heuristic uniform shrinkage factor, (model likelihood-ratio χ^2^ minus degrees of freedom)/likelihood-ratio χ^2^, where values approaching 1 indicate minimal optimism; (ii) a bootstrap-derived calibration slope, where a slope near 1 indicates the prognostic index requires little rescaling; and (iii) a penalized LASSO–Cox model fitted to the full candidate set to confirm that manually selected predictors coincided with those retained under automated penalization. Calibration was further assessed by comparing nomogram-predicted and Kaplan–Meier-observed survival across risk strata [23]. Patients were divided into risk groups by the median nomogram-derived prognostic index and compared by the log-rank test. Baseline CEA was log_10_-transformed. Because the model included both baseline CEA and post-treatment CEA decline, potential collinearity between these terms was assessed by Pearson’s and Spearman’s correlation coefficients, by variance inflation factors (VIFs), and by a sensitivity analysis refitting the model after removing each CEA term in turn. Analyses used Python (lifelines, scikit-survival, and statsmodels) and R; for two-sided tests, *p* < 0.05 was considered significant.

***Additional Analyses:*** The proportional-hazards assumption was assessed for every covariate and globally using scaled Schoenfeld residuals and rank-transformed time-interaction tests, and the functional form of continuous predictors was examined with martingale residuals. The complete candidate-variable set (Table S4) was screened by univariable Cox regression (*p* < 0.10) before multivariable modeling; predictor-selection stability was quantified by refitting the multivariable model in 1000 bootstrap resamples and recording the proportion in which each predictor retained *p* < 0.05. To address the timing of CEA response and potential immortal-time bias, we performed a landmark analysis resetting time zero at the 6-week assessment (restricted to patients who were event-free at the landmark) and a Cox model treating CEA decline as a time-dependent covariate. For hPFS, a competing-risks analysis estimated the cumulative incidence of intrahepatic progression with death as a competing event (Aalen–Johansen) and refitted the predictors using cause-specific and Fine–Gray subdistribution models. A sensitivity analysis restricted to DEB-TACE-treated patients (excluding HAIC monotherapy) was performed, and treatment modality was evaluated as a candidate predictor. Discrimination was compared with a pre-specified reduced reference model (baseline tumor burden and CEA) using optimism-corrected C-indices and likelihood-ratio tests. Calibration-in-the-large was summarized by the calibration intercept and observed/expected ratio at 12 months (OS) and 6 months (hPFS), with calibration points grouped into tertiles of predicted risk. Net clinical benefit was assessed by decision-curve analysis, and the full model equations and baseline survival functions are provided in Table S10.

## 3. Results

### 3.1. Patient Characteristics and Treatment Patterns

The analyzable cohort comprised 63 patients (median age: 57 years; range: 29–82; 67% male). Performance status was preserved in most (ECOG 0–1: 97%). Disease was predominantly left-sided (right-sided primary: 11%), synchronous (89%), and of substantial hepatic burden, with ten or more lesions in 63% and a median largest-lesion diameter of 40 mm. KRAS mutation was present in 49% and BRAF V600E in 8%. Baseline CEA was markedly elevated (median: 84.3 ng/mL). DEB-TACE-based therapy predominated; HAIC monotherapy was uncommon. Baseline characteristics are shown in Table 1.

Among the 63 patients, six were not evaluable for CEA response and one lacked a baseline CEA; one death and five intrahepatic-progression events occurred before the 6-week assessment window (Figure 1). These small numbers limit the potential for immortal-time and selection bias associated with the CEA-response variable.

### 3.2. Survival Overview and Treatment Subgroups

**Figure 2:**
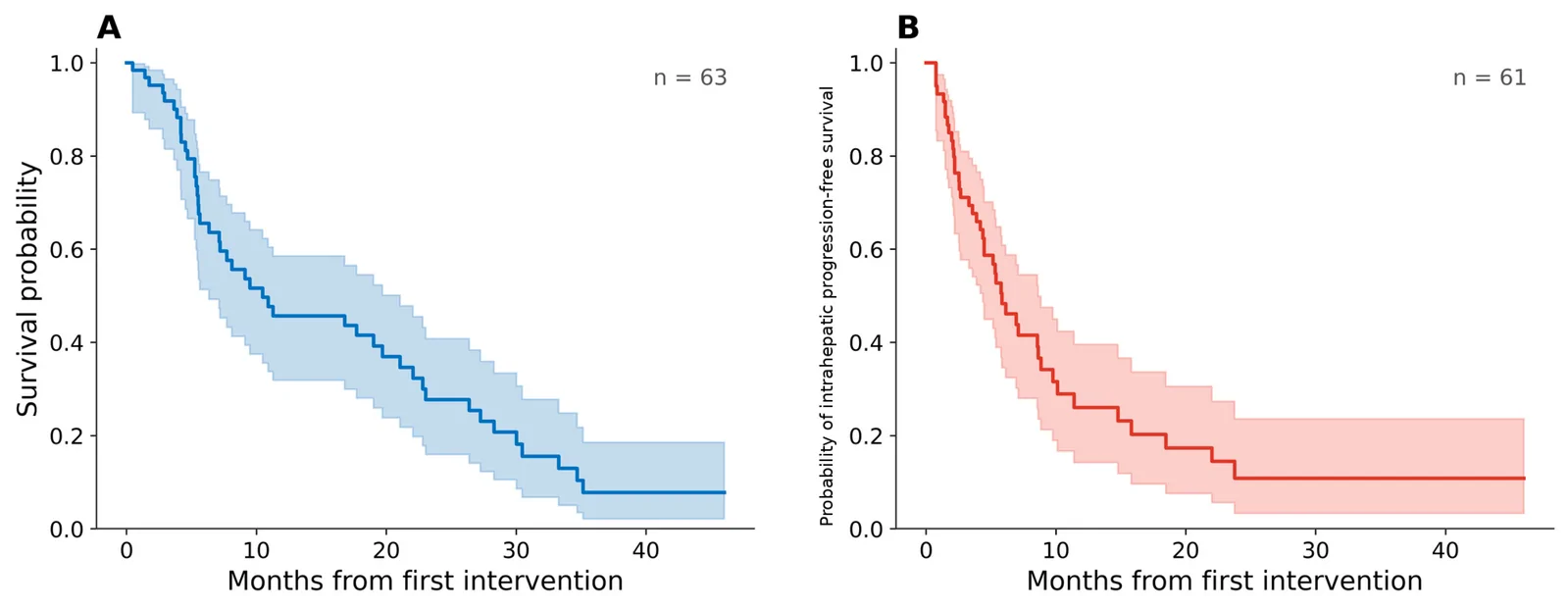
Kaplan–Meier estimates of (**A**) overall survival and (**B**) hepatic progression-free survival for the whole cohort.

At analysis, 44 deaths and 42 intrahepatic-progression events had occurred. Median OS was 10.9 months (95% CI: 7.1–21.1), and median hPFS was 5.8 months (95% CI: 4.2–8.8) (Figure 2). Descriptive subgroup analysis showed a numerically longer median OS with DEB-TACE plus systemic chemotherapy (30.0 months) and HAIC monotherapy (34.7 months) than with DEB-TACE plus HAIC (9.5 months) or DEB-TACE monotherapy (5.5 months) (Figure S1). However, baseline characteristics differed significantly across the four subgroups (Table S1): baseline CEA (*p* = 0.049) and the proportion with high liver tumor burden (*p* = 0.007) were significantly unbalanced. Critically, the longest-surviving subgroups had the lowest tumor burden, with only 12% of the DEB-TACE-plus-systemic subgroup and 40% of the HAIC-monotherapy subgroup presenting with ten or more lesions compared with 76% of the DEB-TACE-plus-HAIC subgroup, which had the shortest survival. These imbalances indicate that the apparent survival differences between subgroups are substantially confounded by baseline selection rather than reflecting comparative treatment efficacy, and are reported as hypothesis-generating only.

### 3.3. Independent Predictors of Survival

Regarding the multivariable Cox regression for OS, four variables were independently associated with outcome: a higher baseline CEA (hazard ratio [HR] per log_10_ unit: 1.59, 95% CI: 1.08–2.32; *p* = 0.017), high liver tumor burden (HR: 2.63, 1.19–5.85; *p* = 0.017), second-line-or-beyond interventional therapy (HR: 3.65, 1.69–7.86; *p* = 0.001), and CEA decline, which was protective (HR: 0.33, 0.16–0.69; *p* = 0.003) (Table 2). Largest-lesion diameter was significant on the univariable analysis but lost independence after adjustment for burden and CEA. Sidedness, KRAS, and BRAF status were not prognostic in this cohort. For hPFS, high liver tumor burden (HR: 3.33, 1.60–6.93; *p* = 0.001), CEA decline (HR: 0.30, 0.15–0.59; *p* < 0.001), and older age (HR: 0.97 per year, 0.95–1.00; *p* = 0.042) were independent predictors; notably, baseline CEA level was not associated with intrahepatic progression (Table 2).

### 3.4. Nomogram Construction and Internal Validation

**Figure 3:**
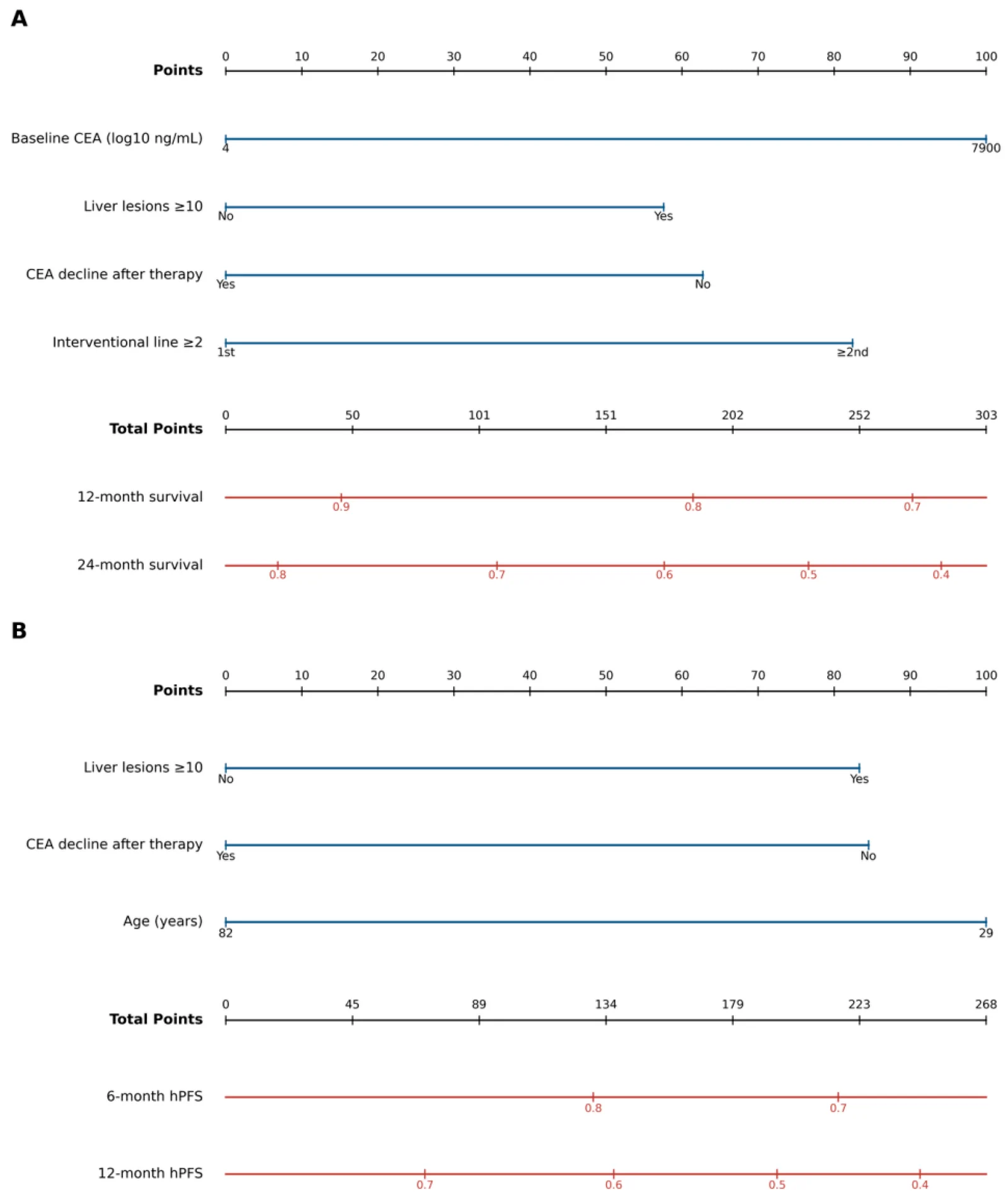
Nomograms predicting (**A**) overall survival and (**B**) hepatic progression-free survival. Points are assigned for each predictor and summed to a total score that maps to predicted survival probabilities. In the hPFS nomogram (**B**), the Age axis is deliberately drawn in descending order (82–29 years) because older age was protective (HR: 0.97); read this axis from right to left when scoring a patient. The Baseline CEA axis (**A**) is on a log10 scale spanning 4–7900 ng/mL; for reference, 10, 100, and 1000 ng/mL fall at approximately 12%, 42%, and 73% of the axis length from the left.

**Figure 4:**
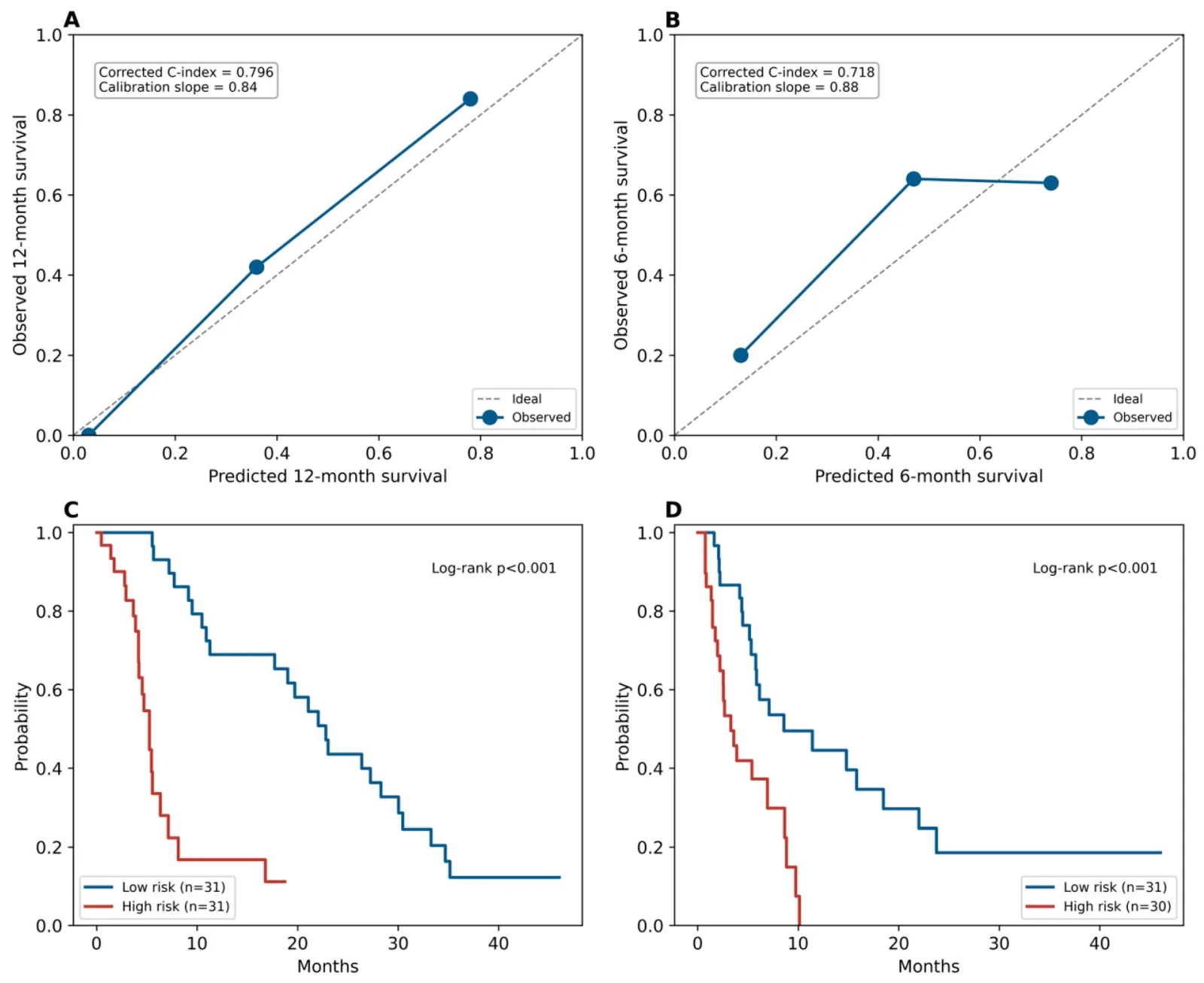
Calibration of the OS nomogram at 12 months (**A**) and the hPFS nomogram at 6 months (**B**), annotated with optimism-corrected C-index and bootstrap calibration slope (calibration points represent tertiles of predicted risk, i.e., three observed-versus-predicted points per panel); and Kaplan–Meier survival curves for the nomogram-defined low- and high-risk groups for OS (**C**) and hPFS (**D**), with log-rank *p* values.

The independent predictors were assembled into separate nomograms for OS and hPFS (Figure 3). Discrimination was good: the OS nomogram achieved an apparent C-index of 0.810 and an optimism-corrected C-index of 0.796; the hPFS nomogram achieved a corrected C-index of 0.718. In repeated 5-fold cross-validation (20 repetitions), the mean C-index was 0.789 (standard deviation: 0.089; 95% CI: 0.60–0.93) for OS and 0.716 (standard deviation: 0.104; 95% CI: 0.45–0.88) for hPFS, closely matching the bootstrap optimism-corrected estimates and confirming model stability across data partitions; the wide intervals reflect the limited sample. Examining overfitting further, the heuristic uniform shrinkage factors were 0.89 (OS) and 0.85 (hPFS), and bootstrap calibration slopes were 0.84 and 0.88—both close to the ideal of 1.0—indicating limited optimism. A LASSO–Cox sensitivity analysis fitted to the full candidate set independently retained the same dominant predictors (high liver burden, CEA decline, baseline CEA, and treatment line), confirming that the selected variables were not artifacts of the screening procedure. Although the OS model included both baseline CEA and post-treatment CEA decline, these terms were only weakly correlated (Pearson’s *r* = 0.13; Spearman’s ρ = 0.13), all variance inflation factors were low (maximum: 1.53, well below the threshold of 5), and removing either CEA term left the other essentially unchanged (CEA-decline HR: 0.33 vs. 0.39; baseline CEA: 1.59 vs. 1.44), indicating that collinearity did not destabilize the hazard-ratio estimates. Calibration showed good agreement between predicted and observed survival at 12 months (OS) and 6 months (hPFS) (Figure 4A,B). Kaplan–Meier survival curves across nomogram-defined risk strata confirmed clear separation between the low- and high-risk groups for both OS and hPFS (Figure 4C,D; both log-rank *p* < 0.001) [23], supporting clinical utility in the absence of external validation. Stratifying by the median nomogram index produced clear separation: high-risk versus low-risk median OS was 5.3 versus 22.8 months, and median hPFS was 3.3 versus 8.6 months (both log-rank *p* < 0.001) (Figure S2). Adherence to TRIPOD items is summarized in Table S3.

***Proportional Hazards and Functional Form:*** The proportional-hazards assumption held for all covariates and globally (global Schoenfeld *p* = 0.431 [OS] and 0.776 [hPFS]; all per-covariate *p* >= 0.13; Table S5), and the linear specification of the continuous predictors was supported.

***Predictor-Selection Stability:*** Across 1000 bootstrap resamples, the retained predictors were significant in a high proportion of samples (OS: treatment line 91.9%, high burden 81.6%, CEA decline 71.5%, and baseline CEA 43.5%; hPFS: CEA decline 97.8%, high burden 92.1%, and age 42.9%), and the LASSO–Cox analysis independently confirmed the dominant predictors (Table S6).

***CEA-Response Timing and Immortal Time:*** The protective association of CEA decline was preserved in the landmark analysis (time zero at 6 weeks; OS HR: 0.406, *p* = 0.017; hPFS HR: 0.223, *p* = 0.001) and when CEA decline was modeled as a time-dependent covariate (OS HR: 0.534, *p* = 0.065; hPFS HR: 0.298, *p* = 0.002), supporting robustness to immortal-time bias; we note, however, that the OS association was attenuated and no longer conventionally significant in the time-dependent model (HR: 0.53, *p* = 0.065), whereas the hPFS association remained significant (Table S7).

***Competing Risks:*** A total of 16 deaths occurred without documented prior intrahepatic progression. The cumulative incidence of intrahepatic progression (death as a competing risk) was 46% at 6 months and 63% at 12 months. Cause-specific associations were preserved (high-burden HR: 4.387; CEA-decline HR: 0.188), whereas on the Fine–Gray subdistribution scale, effect sizes were attenuated (high-burden sHR: 1.251, *p* = 0.498; CEA-decline sHR: 0.603, *p* = 0.117; age sHR: 0.973, *p* = 0.045), reflecting the competing risk of death. Accordingly, the primary Cox hPFS model estimates the cause-specific hazard of documented intrahepatic progression among patients remaining alive and progression-free, rather than the absolute cumulative probability of hepatic progression in the presence of death as a competing event; the hPFS nomogram should be interpreted on this cause-specific basis (Table S8, Figure S3).

***Treatment-Modality Sensitivity:*** Restricting the analysis to DEB-TACE-treated patients (*n* = 58) left the predictors and discrimination essentially unchanged (OS optimism-corrected C-index: 0.805; hPFS: 0.731; Table S9); treatment modality was not independently prognostic (univariable OS HR: 2.841, *p* = 0.086) and did not materially alter the coefficients.

***Incremental Value over Baseline Variables:*** Compared with a reduced reference model (baseline burden and CEA), the full nomograms improved optimism-corrected discrimination (OS: 0.68 to 0.796; hPFS: 0.59 to 0.718) with significant added information (likelihood-ratio *p* &lt; 0.001 for both), confirming that CEA decline, treatment line, and age contribute beyond baseline tumor-burden and CEA information.

***Calibration-in-the-Large and Equations:*** Calibration intercepts were close to zero (OS: −0.151; hPFS: −0.132; observed/expected: 0.91 and 0.921), indicating no systematic miscalibration; calibration points (Figure 4) were grouped into tertiles of predicted risk (three observed-versus-predicted points per panel, consistent with Figure 4). The full linear predictors and baseline survival functions are provided in Table S10.

***Decision-Curve Analysis:*** Both nomograms yielded higher net benefit than treat-all or treat-none strategies across the clinically relevant threshold range (OS: approximately 5–85%; hPFS: approximately 35–85%; Figure S4), supporting potential clinical utility while awaiting external validation.

### 3.5. Safety

Treatment was generally well tolerated. The maximum recorded toxicity was grade 2 in most patients, and grade ≥3 adverse events occurred in only 6% of the cohort. Low-grade post-embolization symptoms (abdominal pain, nausea, and vomiting) were the most frequently recorded events. No treatment-related deaths were attributed to the locoregional procedures (Table S2).

## 4. Discussion

In this real-world cohort treated predominantly with DEB-TACE-based regimens, prognosis was dominated by hepatic tumor burden and by carcinoembryonic antigen, both its baseline level and its dynamic response. We translated these findings into internally validated nomograms for OS and hPFS with good discrimination (optimism-corrected C-indices: 0.796 and 0.718) and calibration, separating patients into risk groups with clinically meaningful survival differences.

The prognostic variables are biologically coherent. Intriguingly, while KRAS (49%) and BRAF V600E (8%) mutations are widely recognized as critical adverse prognostic factors in resectable CRLM settings, they did not reach statistical significance in our univariable survival analysis (Table 2). This phenomenon, though potentially limited by the statistical power of our modest sample size, is not uncommon in real-world cohorts of heavily pre-treated, unresectable patients undergoing arterially directed therapies [15,16]. It suggests that, for patients with advanced disease burden facing acute hepatic progression, the immediate anatomical tumor load (e.g., tumor count ≥ 10) and the early dynamic biochemical response (CEA decline) might exert a more dominant, overriding prognostic weight than baseline molecular alterations alone. These findings highlight the distinct prognostic architecture of locoregionally managed populations compared to surgical or systemic-only cohorts. A high intrahepatic lesion count reflects greater disease bulk and a larger reservoir for progression, and was the only factor independently associated with both endpoints [24,25]. Elevated baseline CEA is an established adverse marker in metastatic colorectal cancer and tracks systemic disease burden, plausibly explaining its stronger association with OS than with intrahepatic control. A decline in CEA after therapy was protective for both endpoints, supporting early biochemical response as a readily available surrogate of benefit [26,27]. The association of a more advanced interventional treatment line with worse OS likely reflects accumulated prior progression and selection of more refractory disease [28,29].

Because the interventional treatment line is a management-related rather than biological variable, it is best interpreted as a surrogate for cumulative disease progression, prior systemic-therapy exposure, acquired resistance, and patient selection, rather than as a modifiable target.

Importantly, the absence of statistical significance for KRAS and BRAF should not be interpreted as evidence that these alterations lack prognostic importance: with 63 patients and only five BRAF V600E-mutated cases, the study is underpowered to detect moderate molecular effects, and these observations are hypothesis-generating.

Our descriptive subgroup analysis illustrates why a formal head-to-head comparison was not appropriate. Subgroups receiving DEB-TACE plus systemic chemotherapy or HAIC monotherapy had longer median survival, but the four subgroups differed significantly in baseline carcinoembryonic antigen (*p* = 0.049) and high-tumor-burden proportion (*p* = 0.007), with the longest-surviving subgroups carrying the lowest disease burden (Table S1). The HAIC-monotherapy subgroup also contained only five patients. Attributing the survival difference to treatment would, therefore, confound treatment effect with baseline selection; we present these comparisons as hypothesis-generating and base our conclusions on the prognostic models, which were developed within the predominantly DEB-TACE-based population rather than across treatment subgroups.

The methodological basis for this caution is well established. When treatment is not randomized, comparing outcomes across treatment subgroups without accounting for baseline differences conflates the effect of treatment with the effect of patient selection; propensity-based or doubly robust methods are advocated as the minimum standard for causal inference from observational oncology data, and naïve subgroup comparisons most reliably mislead when, as here, prognostic factors are unevenly distributed [30]. Empirical work in real-world cancer cohorts has shown that ignoring such selection structures can change estimated outcomes substantially [31]. Our cohort was too small to support credible propensity-based adjustment across four subgroups, which is precisely why we confined formal inference to prognostic modeling within the predominantly DEB-TACE-based population and present the cross-subgroup survival differences as descriptive only.

These observations complement the literature, in which comparisons of DEB-TACE and HAIC have produced mixed results, particularly for response-based endpoints. Rather than asking which modality is superior, we provide individualized prognostic estimates for the patient already receiving DEB-TACE-based therapy—an approach increasingly valued in interventional oncology, where heterogeneous real-world populations limit the transportability of trial-based averages.

Our discrimination compares favorably with published CRLM prognostic models, although direct comparison is limited by differing populations and validation approaches. A prognostic scoring system for resectable CRLM reported a survival C-index of 0.696, with an external-validation C-index of 0.682 for progression-free survival, and identified larger tumors, multiple lesions, RAS mutation, nodal disease, and right-sided primary as adverse factors [32]. Nomograms for recurrent CRLM treated with radiofrequency ablation similarly retained CEA above 5 ng/mL and extrahepatic disease as independent predictors of overall survival [15]. The convergence of our findings (tumor burden and CEA dynamics as dominant prognostic axes) with these independently derived models supports their biological credibility, even though our optimism-corrected OS C-index of 0.796 was obtained by internal validation only and is not directly comparable to externally validated estimates.

These comparisons must be interpreted cautiously: surgically resected and unresectable CRLM populations differ fundamentally in disease biology, baseline prognosis, patient selection, and treatment intent (curative resection versus palliative locoregional therapy), so C-indices and retained predictors are not directly comparable and are presented only as contextual evidence of biological plausibility.

Inflammation-based indices are similarly informative in locoregionally treated CRLM: the lymphocyte-to-monocyte ratio independently predicted survival after radiofrequency ablation for colorectal liver metastases [33], and represents a readily available complement to the tumor-burden and CEA-dynamic factors identified here for future multimodal models.

The discriminatory performance of the two models differed meaningfully, and we interpret them with correspondingly different confidence. Whereas the OS nomogram showed strong discrimination (C-index: 0.796), the hPFS nomogram was only moderate (C-index: 0.718, approaching 0.70), and its clinical generalizability should, therefore, be regarded as more tentative. Predicting intrahepatic progression is intrinsically more challenging than predicting overall survival for several reasons. Local tumor response to arterially directed therapy is heterogeneous and depends on technical factors (superselectivity of catheterization, completeness of embolization, bead size and drug loading, and arterial anatomy) that baseline clinical variables do not capture. The timing and number of repeat interventions also vary between patients in real-world practice, introducing variation in the hazard of intrahepatic progression that is independent of baseline prognosis. Moreover, intrahepatic progression is assessed radiologically and is subject to imaging-interval and interpretation variability, whereas death is an unambiguous endpoint. Overall survival additionally integrates extrahepatic disease and systemic-treatment effects, which may render it more strongly and stably associated with baseline tumor burden and CEA. We therefore recommend that the hPFS nomogram be used cautiously—primarily for hypothesis generation and for flagging patients at higher risk of early intrahepatic failure rather than as a definitive predictive tool—and we regard its external validation as a particular priority. Consistent with the competing-risks analysis, the hPFS nomogram predicts the cause-specific hazard of intrahepatic progression conditional on remaining alive and progression-free, not the absolute cumulative incidence of hepatic progression, and should be interpreted on that basis.

To illustrate clinical use, consider a patient beginning second-line DEB-TACE with a baseline CEA of 200 ng/mL, ten or more hepatic lesions, and no subsequent CEA decline. On the overall-survival nomogram, this combination accrues a high total score, placing the patient in the high-risk stratum, whose median overall survival was 5.3 months. Such an estimate could prompt earlier discussion of systemic intensification, closer imaging surveillance, or supportive-care planning. Conversely, a first-line patient with a baseline CEA of 8 ng/mL, fewer than ten lesions, and a post-treatment CEA decline accrues a low score, corresponding to the low-risk stratum with a median overall survival of 22.8 months, supporting continuation of the current locoregional strategy. The nomograms, thus, translate routinely available variables into individualized, actionable estimates at the point of care.

Our clinical model also points to clear directions for refinement. Image-based quantitative analysis is an obvious complement: radiomics models built on contrast-enhanced CT or MRI have predicted overall survival and lesion-level response in patients receiving irinotecan-loaded chemoembolization in the prospective CIREL cohort and in radioembolization series [34,35], and recent reviews highlight radiomics and radiogenomics as natural complements to clinical variables for CRLM [36]. Early three-dimensional volumetric response on cross-sectional imaging after intra-arterial therapy has likewise been associated with subsequent survival [37], suggesting that integrating early imaging dynamics with the biochemical response we identified could sharpen prediction, particularly for the harder endpoint of intrahepatic progression. Molecular context may add further value: the liver microenvironment in microsatellite-stable colorectal cancer is recognized as a major contributor to immune-checkpoint resistance [38], and adjuvant combinations of immunotherapy and chemotherapy after curative-intent resection of CRLM have shown improved survival in a propensity-matched analysis [39]. Incorporating radiomic, dynamic-imaging, and extended molecular features into externally validated, multimodal models is a logical next step for individualized prognostication in this population. Furthermore, our nomogram-defined risk stratification showed clear survival separation between the low- and high-risk groups (Figure 4C,D; both log-rank *p* < 0.001), supporting the models’ potential clinical utility. For instance, patients identified as “high-risk” by our model, who exhibit a poor median overall survival of only 5.3 months, should be considered candidates for immediate multidisciplinary intervention. In a clinical “Medicine + X” framework, this quantified high-risk status could justify shifting from standard routines to intensified systemic regimens, early integration of targeted or immune-checkpoint therapies, or significantly shortened imaging follow-up intervals, thereby shifting the paradigm from reactive management to proactive personalized oncology.

These proposed actions are hypotheses supported by discrimination, calibration, and decision-curve (net-benefit) analysis but not yet by prospective impact studies, and require external validation before routine clinical use.

Several limitations must be emphasized, the foremost being sample size and the absence of external validation. With 63 patients and 44 deaths, any multivariable model carries a risk of overfitting. We addressed this directly: events-per-variable ratios (approximately 11 and 14) met conventional thresholds, and the shrinkage factors (0.89 and 0.85) and calibration slopes (0.84 and 0.88) indicated mild optimism, with the LASSO–Cox analysis independently selecting the same predictors. These convergent results increase confidence that the models capture genuine signal but cannot replace external validation; the corrected metrics guard against, but do not eliminate, reduced performance in new populations, and calibration slopes below 1.0 imply that predictions may need recalibration externally [31]. We therefore regard the nomograms as internally validated, hypothesis-strengthening tools requiring external, ideally multicenter, confirmation. Additional limitations include the single-center retrospective design, the small HAIC-monotherapy subgroup, the absence of radiomic and extended molecular features, and limited follow-up in a subset of patients.

Established prognostic domains not captured here (extrahepatic disease burden, liver-function parameters, extended molecular profiling, detailed prior systemic-therapy history, radiomic/imaging features, and technical procedural variables) may reduce the biological completeness of the model and could contribute to the non-significance of individual molecular markers; several omissions reflect sample-size and data-availability constraints. The heterogeneity of treatment pathways is a further limitation, although the retained predictors are patient- and disease-level and remained stable in the DEB-TACE-restricted analysis.

## 5. Conclusions

In real-world DEB-TACE-treated unresectable CRLM, hepatic tumor burden and CEA level and dynamics were the principal determinants of overall survival and hepatic progression-free survival. The internally validated nomograms derived from these factors offer individualized prognostic estimates and clear risk stratification, and may inform multidisciplinary decision-making once externally validated. Because the models rely only on variables routinely obtained during interventional care, they can be applied at the bedside without additional cost or specialized assays.

Because the models are internally validated only, they should be regarded as hypothesis-strengthening tools requiring external, ideally multicenter, validation and prospective impact evaluation before routine clinical use.

## Figures and Tables

**Table 1:** Baseline characteristics of the analyzable cohort.

Characteristic	Value (*n* = 63)
Age, years, median (range)	57 (29–82)
Male sex, no. (%)	42 (67)
ECOG performance status 0–1, no. (%)	61 (97)
Right-sided primary, no. (%)	7 (11)
KRAS mutation, no. (%)	31 (49)
BRAF V600E mutation, no. (%)	5 (8)
Synchronous metastases, no. (%)	56 (89)
High liver tumor burden (≥10 lesions), no. (%)	40 (63)
Liver involvement >50%, no. (%)	6 (10)
Largest lesion, mm, median (range)	40 (9–200)
Baseline CEA, ng/mL, median (range)	84.3 (2.7–6744)
CEA decline after therapy, no. (%)	33 (52)
Interventional therapy second-line or beyond, no. (%)	35 (56)
Primary tumor resected, no. (%)	42 (67)

Abbreviations: BRAF, B-Raf proto-oncogene; CEA, carcinoembryonic antigen; ECOG, Eastern Cooperative Oncology Group; KRAS, Kirsten rat sarcoma viral oncogene homolog.

**Table 2:** Univariable and multivariable Cox regression for overall survival and hepatic progression-free survival.

Variable	Univariable HR (95% CI)	*p* ^a^	Multivariable HR (95% CI)	*p* ^a^
***Overall survival***				
Baseline CEA (per log_10_)	1.67 (1.23–2.26)	0.001	1.59 (1.08–2.32)	0.017
High liver burden (≥10)	2.44 (1.30–4.61)	0.006	2.63 (1.19–5.85)	0.017
CEA decline	0.46 (0.25–0.86)	0.015	0.33 (0.16–0.69)	0.003
Second-line or beyond	3.23 (1.67–6.25)	<0.001	3.65 (1.69–7.86)	0.001
Largest lesion (per mm)	1.01 (1.00–1.02)	0.007	NA	NA
Right-sided primary	0.99 (0.35–2.79)	0.978	NA	NA
KRAS mutation	1.20 (0.66–2.17)	0.555	NA	NA
***Hepatic progression-free survival***				
High liver burden (≥10)	1.99 (1.04–3.83)	0.039	3.33 (1.60–6.93)	0.001
CEA decline	0.41 (0.22–0.76)	0.005	0.30 (0.15–0.59)	<0.001
Age (per year)	0.98 (0.96–1.00)	0.097	0.97 (0.95–1.00)	0.042
Second-line or beyond	1.75 (0.93–3.28)	0.081	NA	NA
Baseline CEA (per log_10_)	1.14 (0.84–1.55)	0.404	NA	NA

^a^ Two-sided *p* value from Cox proportional-hazards regression. NA indicates a variable not retained in the multivariable model; age, per additional year (the hPFS age HR of 0.97 means each extra year lowers the intrahepatic-progression hazard by ~3%); largest lesion, per 1 mm increase. Abbreviations: CEA, carcinoembryonic antigen; CI, confidence interval; HR, hazard ratio; KRAS, Kirsten rat sarcoma viral oncogene homolog.

## Data Availability

The data presented in this study are available from the corresponding author upon reasonable request, subject to institutional and ethical approval.

## Supplementary Materials

The following supporting information can be downloaded at: https://www.mdpi.com/article/10.3390/curroncol33090497/s1. Figure S1: Descriptive overall survival by treatment subgroup (hypothesis-generating; small, unbalanced subgroups); Figure S2: Kaplan–Meier curves for overall survival (A) and hepatic progression-free survival (B) by nomogram-defined risk group (median split of the prognostic index); Figure S3: Cumulative incidence of intrahepatic progression with death as a competing risk; Figure S4: Decision-curve analysis; Table S1: Baseline characteristics across the four treatment subgroups, with *p* values (Kruskal–Wallis or χ^2^); Table S2: Adverse events graded by CTCAE v5.0; Table S3: Adherence to the TRIPOD reporting checklist (key items); Table S4: Complete set of candidate predictors considered; Table S5: Proportional-hazards assessment (scaled Schoenfeld residuals; rank-transformed time); Table S6: Predictor-selection stability across 1000 bootstrap resamples; Table S7: Immortal-time sensitivity analyses for CEA decline; Table S8: Competing-risks analysis for hepatic progression-free survival (death as competing event); Table S9: Sensitivity analysis restricted to DEB-TACE-treated patients (*n* = 58; HAIC monotherapy excluded); Table S10: Model equations and baseline survival functions; Table S11: Incremental value: full nomogram versus reduced reference model (baseline burden + CEA); Table S12: Dose–response sensitivity analysis: ordinal liver tumor-burden level (low ≤ 4; intermediate 5–9; high ≥ 10 lesions).

## Abbreviations

The following abbreviations are used in this manuscript:

BRAF	B-Raf proto-oncogene
CEA	Carcinoembryonic antigen
CI	Confidence interval
C-index	Concordance index
CRLM	Colorectal liver metastases
CTCAE	Common Terminology Criteria for Adverse Events
DEB-TACE	Drug-eluting bead transarterial chemoembolization
ECOG	Eastern Cooperative Oncology Group
HAIC	Hepatic arterial infusion chemotherapy
hPFS	Hepatic progression-free survival
HR	Hazard ratio
KRAS	Kirsten rat sarcoma viral oncogene homolog
LASSO	Least absolute shrinkage and selection operator
OS	Overall survival
TRIPOD	Transparent Reporting of a multivariable prediction model for Individual Prognosis Or Diagnosis
VIF	Variance inflation factor
