# Supplementary material for: Treatment Patterns and Prognostic Nomograms for Overall Survival and Hepatic Progression-Free Survival in Unresectable Colorectal Liver Metastases Treated with Drug-Eluting Bead Chemoembolization: A Single-Center Study
Source: Curr Oncol. 2026 Aug 22;33(9):497. doi: 10.3390/curroncol33090497 (PMC13605775; doi:10.3390/curroncol33090497)
Supplement: Supplementary file 1 [file curroncol-33-00497-s001.zip › curroncol-4454547-supplementary.pdf]

Supplementary Materials

Prognostic nomograms for OS and hPFS in DEB-TACE-treated unresectable CRLM

All values were computed from the study dataset (OS analytic cohort n = 63, 44 deaths; hPFS analytic cohort n = 61, 42 intrahepatic-progression events).

Table S1. Baseline characteristics across the four treatment subgroups.

Characteristic	DEB-TACE mono (n=15)	DEB-TACE + HAIC (n=33)	DEB-TACE + systemic (n=8)	HAIC mono (n=5)	*P* [a]
Age, y, median	59	57	52	53	0.77
Male sex, no. (%)	10 (67)	24 (73)	3 (38)	3 (60)	0.31
Right-sided primary, no. (%)	1 (7)	4 (12)	2 (25)	0 (0)	0.49
KRAS mutation, no. (%)	4 (27)	19 (58)	5 (62)	2 (40)	0.19
Synchronous disease, no. (%)	12 (80)	29 (88)	8 (100)	5 (100)	0.43
Baseline CEA, ng/mL, median	74	266	11	27	0.049
High liver burden (≥10), no. (%)	10 (67)	25 (76)	1 (12)	2 (40)	0.007
Liver involvement >50%, no. (%)	1 (7)	5 (15)	0 (0)	0 (0)	0.45
Largest lesion, mm, median	60	41	41	18	0.13
Second-line or beyond, no. (%)	11 (73)	17 (52)	5 (62)	1 (20)	0.18
Median overall survival, mo	5.5	9.5	30.0	34.7	NA

a P values from the Kruskal–Wallis test (continuous variables) or the χ^2 test (categorical variables) across the four subgroups. Bolded values denote statistically significant baseline differences ($P < 0.05$). Two patients receiving other combinations or conventional TACE were not included in this four-group comparison. Abbreviations: CEA, carcinoembryonic antigen; DEB-TACE, drug-eluting bead transarterial chemoembolization; HAIC, hepatic arterial infusion chemotherapy; KRAS, Kirsten rat sarcoma viral oncogene homolog.

Table S2. Adverse events graded by CTCAE v5.0.

Adverse event category	no. (%), n = 63
Any adverse event recorded	41 (65)
Maximum grade 2	37 (59)
Maximum grade 3	2 (3)
Maximum grade 4	2 (3)
Grade ≥3 overall	4 (6)
Post-embolization symptoms (pain, nausea, vomiting)	Most frequent low-grade events

Table S3. Adherence to the TRIPOD reporting checklist (key items).

TRIPOD item	Location
Source of data; eligibility criteria	Methods
Outcome definitions (OS, hPFS)	Methods
Predictors and definitions	Methods; Table 1
Sample size and events	Results
Model-building procedure	Methods
Model presentation (nomogram)	Figure 3
Discrimination (C-index)	Results
Internal validation (bootstrap, shrinkage, slope)	Methods; Results
Calibration	Figure 4A,B
Limitations incl. no external validation	Discussion

Table S4. Complete set of candidate predictors considered.

Candidate variable	Type	Screening result
Age	Continuous	Entered (hPFS)
Sex	Binary	Screened out
ECOG performance status (0-1 vs 2)	Binary	Screened out
Primary tumor sidedness (right vs left)	Binary	Screened out
KRAS mutation	Binary	Screened out
BRAF V600E mutation	Binary	Screened out
Synchronous vs metachronous	Binary	Screened out
High liver tumor burden (>=10 lesions)	Binary	Retained (OS, hPFS)
Liver involvement >50%	Binary	Screened out
Largest lesion diameter (mm)	Continuous	Univariable only (OS)
Baseline CEA (log10)	Continuous	Retained (OS)
Post-treatment CEA decline	Binary	Retained (OS, hPFS)
Interventional treatment line (>=2nd)	Binary	Retained (OS)
Primary tumor resection	Binary	Screened out
Treatment modality (DEB-TACE-based vs HAIC)	Categorical	Screened out (see Table S9)

Univariable Cox screening at P < 0.10; final models constrained by events-per-variable (4 for OS, 3 for hPFS). LASSO-Cox retained the same dominant predictors.

Table S5. Proportional-hazards assessment (scaled Schoenfeld residuals; rank-transformed time).

Model / covariate	P value
OS: baseline CEA (log10)	0.502
OS: high liver burden	0.443
OS: treatment line	0.482
OS: CEA decline	0.131
OS: GLOBAL	0.431
hPFS: high liver burden	0.501
hPFS: CEA decline	0.488
hPFS: age	0.68
hPFS: GLOBAL	0.776

All P > 0.05 indicate no evidence of proportional-hazards violation.

Table S6. Predictor-selection stability across 1000 bootstrap resamples.

Model / predictor	Significant in resamples (bootstrap HR, 95% percentile interval)
OS: treatment line	91.9% (HR 4.41, 1.74-14.23)
OS: high liver burden	81.6% (HR 3.82, 1.45-14.22)
OS: CEA decline	71.5% (HR 0.36, 0.12-0.72)
OS: baseline CEA	43.5% (HR 1.48, 0.94-2.29)
hPFS: CEA decline	97.8% (HR 0.18, 0.06-0.42)
hPFS: high liver burden	92.1% (HR 4.2, 1.8-15.04)
hPFS: age	42.9% (HR 0.97, 0.94-1)

Multivariable model refitted in each resample; "significant" = P < 0.05. Borderline frequencies for baseline CEA and age mirror their borderline P values in the primary model.

Table S7. Immortal-time sensitivity analyses for CEA decline.

Analysis	Endpoint	HR (P)
Landmark at 6 weeks (OS n=57)	OS	0.406 (P=0.0171)
Landmark at 6 weeks (hPFS n=53)	hPFS	0.223 (P=0.001)
Time-dependent covariate	OS	0.534 (P=0.0651)
Time-dependent covariate	hPFS	0.298 (P=0.0022)

The protective association of CEA decline persists after resetting time zero at the assessment (landmark) and when modelled as time-dependent, indicating it is not an immortal-time artefact.

Table S8. Competing-risks analysis for hepatic progression-free survival (death as competing event).

Predictor	Cause-specific HR (P)	Fine-Gray sHR (P)
High liver burden	4.387 (P=0.0005)	1.251 (P=0.4984)
CEA decline	0.188 (P=0.0001)	0.603 (P=0.1166)
Age	0.975 (P=0.0603)	0.973 (P=0.0447)

Deaths without prior intrahepatic progression: 16. Cumulative incidence of intrahepatic progression: 46% at 6 months, 63% at 12 months (Figure S3). Cause-specific effects (used by the nomogram) are preserved; Fine-Gray subdistribution effects attenuate owing to the competing risk of death, supporting cautious interpretation of the hPFS model.

Table S9. Sensitivity analysis restricted to DEB-TACE-treated patients (n = 58; HAIC monotherapy excluded).

Predictor	OS HR (P)	hPFS HR (P)
High liver burden	3.814 (P=0.0035)	3.806 (P=0.0021)
CEA decline	0.339 (P=0.0052)	0.224 (P=0.0005)
Baseline CEA (log10)	1.426 (P=0.0658)	-
Treatment line (>=2nd)	3.297 (P=0.0026)	-
Age	-	0.977 (P=0.0806)

Optimism-corrected C-index in the restricted cohort: OS 0.805, hPFS 0.731 (vs 0.809 / 0.744 in the full cohort). Treatment modality was not independently prognostic (univariable OS HR 2.841, P=0.0856).

Table S10. Model equations and baseline survival functions.

Predicted survival: $S(t) = S0(t) ^{\wedge} \exp(LP - \bar{LP})$, where LP is the linear predictor below and terms in [ ] are coded 1/0.

OS linear predictor: $LP = 0.3662 \cdot \log10(CEA) + 1.2655 \cdot [\geq 10 \text{ lesions}] + 1.4027 \cdot [\text{2nd-line+}] + (-0.9425) \cdot [\text{CEA decline}]$

S0(12 mo) = 0.3699 at mean covariates; $\bar{LP} = 1.8243$. (Baseline survival at covariate zero: 0.8518.)

hPFS linear predictor: $LP = 1.4786 \cdot [\geq 10 \text{ lesions}] + (-1.6699) \cdot [\text{CEA decline}] + (-0.0251) \cdot \text{age(years)}$

S0(6 mo) = 0.3642 at mean covariates; $\bar{LP} = -1.3781$. (Baseline survival at covariate zero: 0.0182.)

Additional baseline survival for the horizons shown in Figure 3: OS S0(24 mo) = 0.067; hPFS S0(12 mo) = 0.0358 (at mean covariates). These, with the linear predictors above, reproduce all survival probabilities displayed in Figure 3.

Coefficients are ln(HR) from the multivariable Cox models fitted to the analysis dataset; baseline survival estimated by the Breslow method. These allow exact reproduction and external validation.

Table S11. Incremental value: full nomogram versus reduced reference model (baseline burden + CEA).

Endpoint	Reduced C-index (corrected)	Full C-index (corrected)	Likelihood-ratio test
OS	0.676	0.796	chi2=24.44, P<0.001
hPFS	0.589	0.718	chi2=23.57, P<0.001

Full-model optimism-corrected C-indices are the principal values (0.796 OS, 0.718 hPFS); reduced-model values from the same 1000-bootstrap procedure. (An earlier draft reported 0.809/0.744 from a separate bootstrap run; values are now harmonized.)

Adding CEA decline, treatment line (OS) and age (hPFS) significantly improves discrimination beyond readily available baseline variables.

Table S12. Dose-response sensitivity analysis: ordinal liver tumor-burden level (Low <=4, Mid 5-9, High >=10 lesions).

Endpoint	HR per level (95% CI)	P value	C-index	n
OS	1.66 (1.11-2.47)	0.013	0.637	61
hPFS	1.49 (0.98-2.28)	0.061	0.581	61

Univariable Cox regression with burden entered as an ordinal 0/1/2 term (n = 61; two patients with unknown burden excluded). The graded association supports the pre-specified ten-lesion dichotomy; exact continuous modelling was precluded because lesion counts were frequently recorded as ranges.

Figure S1. Descriptive overall survival by treatment subgroup (hypothesis-generating).

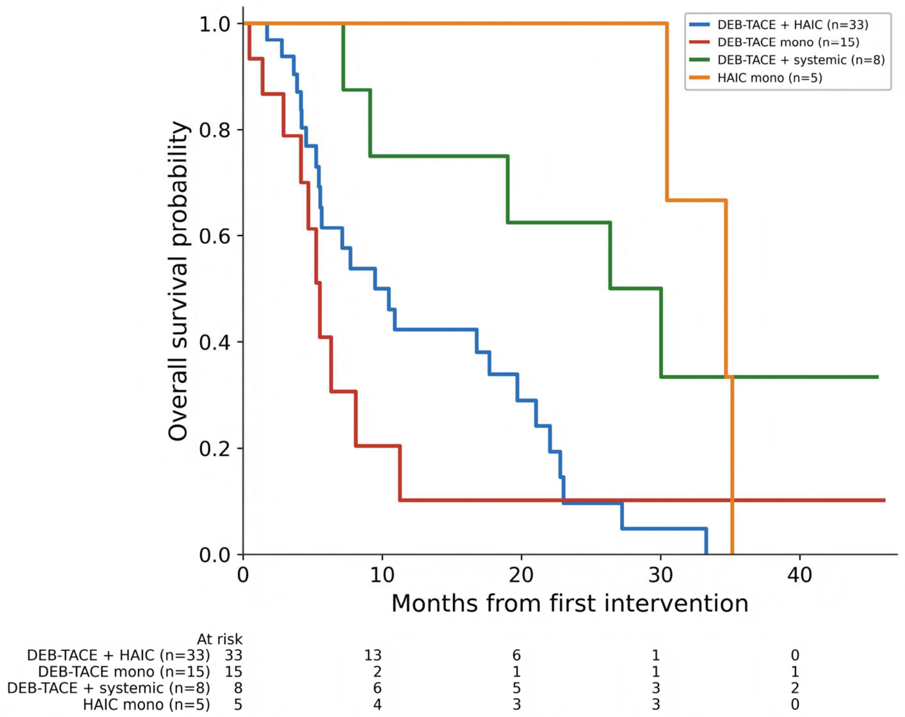

Kaplan-Meier overall survival for the four real-world treatment subgroups, with the at-risk table below. Subgroups were small and imbalanced (Table S1); comparisons are descriptive only.

Figure S2. Kaplan-Meier survival by nomogram-defined risk group.

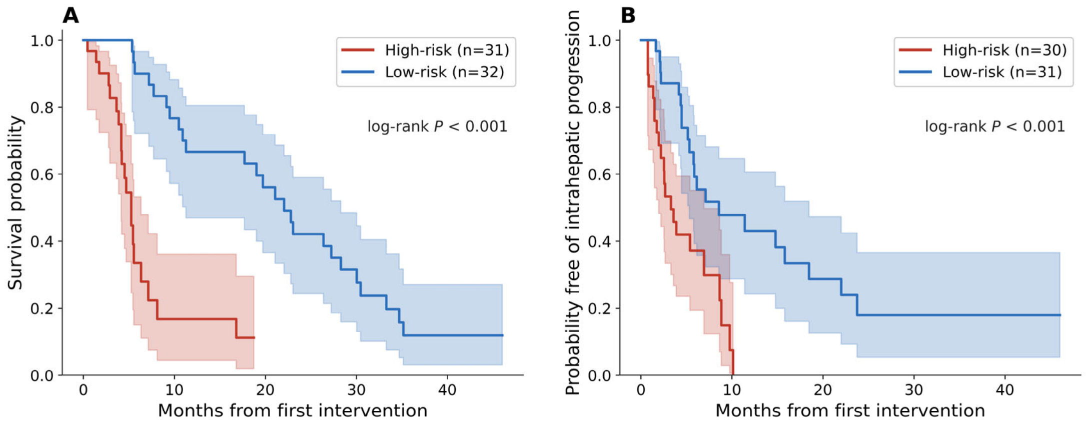

Overall survival (A) and hepatic progression-free survival (B) for the low- and high-risk groups defined by the median nomogram prognostic index (log-rank P < 0.001 for both).

Figure S3. Cumulative incidence of intrahepatic progression with death as a competing risk.

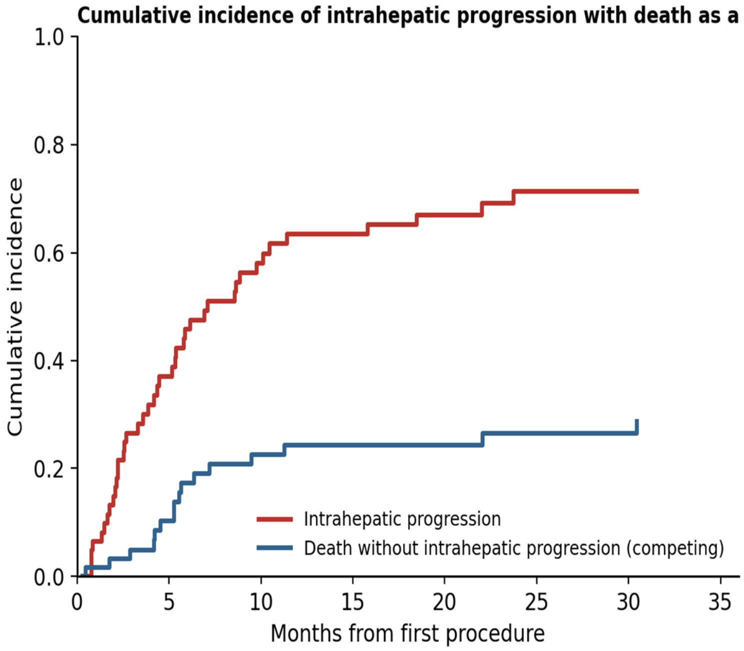

Aalen-Johansen estimates. Intrahepatic progression (red) and death without intrahepatic progression (blue).

Figure S4. Decision-curve analysis.

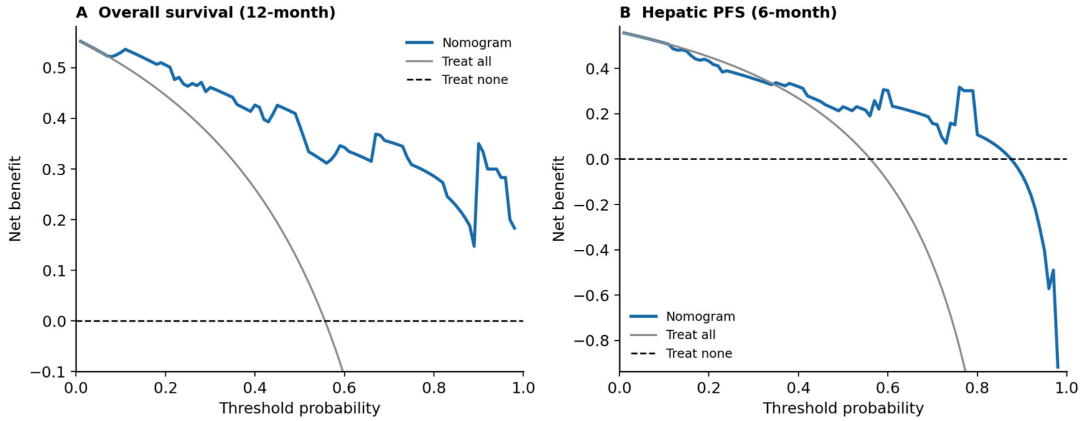

Net benefit of the OS nomogram at 12 months (A) and the hPFS nomogram at 6 months (B) versus treat-all and treat-none strategies.
